# Effectiveness and safety of ginkgo biloba preparations in the treatment of Alzheimer's disease: A systematic review and meta-analysis

**DOI:** 10.3389/fnagi.2023.1124710

**Published:** 2023-03-07

**Authors:** Dawei Li, Jinlong Ma, Baojian Wei, Shuang Gao, Yanmei Lang, Xueying Wan

**Affiliations:** ^1^School of Nursing, Shandong First Medical University & Shandong Academy of Medical Sciences, Taian, Shandong, China; ^2^School of Nursing, Yanbian University, Yanji, Jilin, China; ^3^School of Continuing Education, Shandong First Medical University & Shandong Academy of Medical Sciences, Taian, Shandong, China

**Keywords:** donepezil hydrochloride, ginkgo biloba preparations, Alzheimer's disease, meta-analysis, systematic review

## Abstract

**Objective:**

To conduct a meta-analysis of the effectiveness and safety of ginkgo biloba preparations combined with donepezil hydrochloride vs. donepezil for the treatment of Alzheimer's disease (AD).

**Methods:**

Three English databases (Cochrane Library, PubMed, EMBASE), and four Chinese databases [the China National Knowledge Infrastructure (CKNI), the Chinese Biomedical Literature database (CBM), the Chongqing VIP database, and WANFANG DATA)] were manually searched for literature published from the respective dates of inception of the databases to December 2022. The randomized controlled trials (RCTs) of ginkgo biloba preparations with donepezil hydrochloride vs. donepezil for the treatment of AD were included. Relevant literature was screened, and the data in the included studies were extracted for quality assessment according to the Risk of bias tool. The RevMan 5.3 software was used for meta-analysis.

**Results:**

A total of 1,642 participants were enrolled in the 18 RCTs. Of these, 842 were in the experimental group (ginkgo biloba preparations combined with donepezil hydrochloride) and 800 were in the control group (donepezil). The overall methodological quality of the included RCTs is poor due to the high risks of blindness and allocation concealment. The meta-analysis results showed statistically significant differences in several outcomes including Risk Ratio (RR) in change for clinical effectiveness rate (1.23, 95% CI 1.13, 1.34, *P* < 0.00001), mean difference (MD) in change for Mini-Mental State Examination score (3.02, 95% CI 2.14, 3.89, *P* < 0.00001), Activity of Daily Living Scale score (−4.56, 95% CI −5.09, −4.03, *P* < 0.00001), Hasegawa Dementia Scale score (2.04, 95% CI 1.74, 2.34, *P* < 0.00001), Montreal Cognitive Assessment score (2.38, 95% CI 0.72, 4.06, *P* = 0.005), between the experimental and control groups. But there is no statistically significant difference in change for adverse reaction (0.91, 95% CI 0.58, 1.42, *P* = 0.69).

**Conclusion:**

Ginkgo biloba preparations plus donepezil can improve clinical effectiveness rate and vocabulary memory outcomes. However, more relevant high-quality RCTs are needed in the future to validate these results.

**Systematic review registration:**

Identifier CRD42022378970.

## 1. Introduction

Alzheimer's disease (AD) is a progressive and insidious neurodegenerative disease common among the elderly (Klyucherev et al., 2022). With economic development and advanced medical care, there has been a significant increase in human life expectancy hence an increased prevalence of AD (Huang et al., 2019). The main clinical manifestations of AD are diminished intelligence, poor memory, reduced language ability, absent-mindedness, difficulty in distinguishing things, and varying degrees of personality changes. Late initiation of intervention and treatment leads to a serious impact on the patient and causes a significant burden on the family and society. Currently, there are many clinical treatment regimens for AD but limited curative effects, hence the need for further exploration of more interventions. Thus, a comparison of the effectiveness of traditional drugs, represented by donepezil, and traditional Chinese medicines, represented by ginkgo, for the treatment of AD has become an area of interest (Barten and Albright, 2008). Donepezil hydrochloride, a typical acetylcholinesterase inhibitor, also has certain negative effects such as diarrhea, muscle spasm, and other symptoms, which makes some patients urgently need other drugs for treatment whereas (da Silva et al., 2011), ginkgo can improve brain metabolism and protect against ischemia and hypoxia. Ginkgo biloba extract is extensively used in the management of numerous diseases, including cardiovascular disease and coronary heart disease (Shaito et al., 2020; Xie et al., 2022). Besides, a meta-analysis indicated that ginkgo biloba extract could effectively improve the cognitive function of patients with vascular cognitive impairment (Zhan et al., 2021; García-Alberca et al., 2022). However, Tan et al. (2015) did not establish the efficiency of ginkgo biloba treatment in patients with ADAlthough both ginkgo preparation and donepezil can treat AD, no meta-analysis studies have explored the effectiveness and safety of ginkgo preparation combination and donepezil alone. Therefore, we conducted a meta-analysis of the available trials on the effectiveness and safety of ginkgo biloba preparations combined with donepezil hydrochloride vs. donepezil for the treatment of AD.

## 2. Methods

We registered a standard protocol, developed before study selection, for all steps of this meta-analysis on the PROSPERO platform, and the approval number for registration is CRD42022378970. In addition, we followed the PRISMA checklist (Page et al., 2021) reporting guideline to present this meta-analysis.

### 2.1. Search strategy

Three English databases (Cochrane Library, PubMed, EMBASE) and four Chinese databases [the China National Knowledge Infrastructure (CKNI), Chinese Biomedical Literature database (CBM), the Chongqing VIP database, and WANFANG DATA were manually searched for literature published from dates of the inception of the databases to 24 November, 2022 (Beijing time). In order to comprehensively search and obtain relevant literature, a manual search was carried out using the following search terms and their variants: extract of ginkgo biloba, ginkgo leaf, and Alzheimer's disease. The detailed search strategy is provided in Supplementary Tables 1–3.

### 2.2. Inclusion and exclusion criteria

#### 2.2.1. Inclusion criteria

(1) Study population: patients diagnosed with Alzheimer's disease according to internationally accepted diagnostic criteria DSM-IV-R (Diagnostic and Statistical Manual of Mental Disorder, 4th edition, Revised) and ICD-10 (International Classification of Diseases, Tenth Revision) diagnostic criteria; National Institute on Aging-Alzheimer's Association, NIA-AA; (2) study type: randomized controlled trial; (3) intervention: administration of ginkgo combined with donepezil hydrochloride in the experimental group and donepezil alone in the control group; (4) clinical outcomes: primary outcome including clinical effectiveness rate and Mini-Mental State Examination (MMSE) scores; secondary outcome including Activity of Daily Living Scale score (ADL), Hasegawa Dementia Scale score (HDS), Montreal Cognitive Assessment score (MCoA), and adverse events as safety outcomes.

#### 2.2.2. Exclusion criteria

(1) Meeting abstracts, letter to editorials; (2) duplicate publication; (3) incomplete data; (4) no interested outcomes.

### 2.3. Study selection

We import the initial records retrieved from these databases into NoteExpress reference management software and use the software's duplicate checking function in combination with manual screening to eliminate duplication. Two reviewers (Ma Jinlong and Gao Shuang) further read the title and abstract of the initial records, respectively, and excluded irrelevant records. Then, the rest of the full text was read, and the final literature for meta-analysis determined according to the pre-established inclusion and exclusion criteria. Any disputes were resolved through group discussion.

### 2.4. Data extraction

A self-generated information extraction form was also used to collect details of the included studies. The extracted information included title, corresponding author, time of publication, elements of methodological quality evaluation, age, sample size, time, and specific interventions administered to the patients in the treatment and control groups.

### 2.5. Assessment of methodological quality

Two authors (Lang Yanmei and Meng Li) simultaneously assessed the methodological quality of included RCTs, according to the Cochrane Handbook for Systematic Reviews of Interventions (Higgins et al., 2011). The evaluated items were as follows: random sequence generation, allocation concealment, blinding of participants and personnel, blinding of outcome assessment, incomplete outcome data, selective reporting, and other biases. The disagreements were resolved through discussion with the third reviewer (Wei Baojian).

### 2.6. Quality of evidence

We evaluated the quality of evidence for primary and secondary outcomes according to the Grading of Recommendations Assessment, Development, and Evaluation (GRADE) (Balshem et al., 2011) for risk of bias, inconsistency, indirectness, imprecision, and publication bias. The quality of evidence was classified as high, moderate, low, or very low. Summary tables were constructed using the GRADE Profiler (https://gradepro.org/) (Guyatt et al., 2011).

### 2.7. Statistical analysis

Qualitative data were expressed as risk ratio (RR) for effect sizes and weighted mean difference (MD) or standardized mean difference, with a significance level of α = 0.05 and 95% confidence interval (CI). Heterogeneity tests were based on *P*-values obtained from Q tests combined with the *I*^2^ statistic. When the results of the heterogeneity test were *P* > 0.1 and *I*^2^ ≤ 50%, the heterogeneity among the included studies was considered to be relatively small and the meta-analysis was performed using a fixed-effects model. When the results of the heterogeneity test were *P* ≤ 0.1 and *I*^2^ > 50%, indicating that the heterogeneity among the studies was relatively large, a random-effects model was used for the meta-analysis. When the number of suitable RCTs meets the requirement of 10 or more, the inverted funnel plot would be made to evaluate the influence of publication bias on our results. For the outcome indicators with high heterogeneity, the sensitivity analysis was carried out by removing individual original studies one by one. Meta-analysis of the data was performed using RevMan 5.3 software.

## 3. Results

### 3.1. Selection results

**Figure 1:**
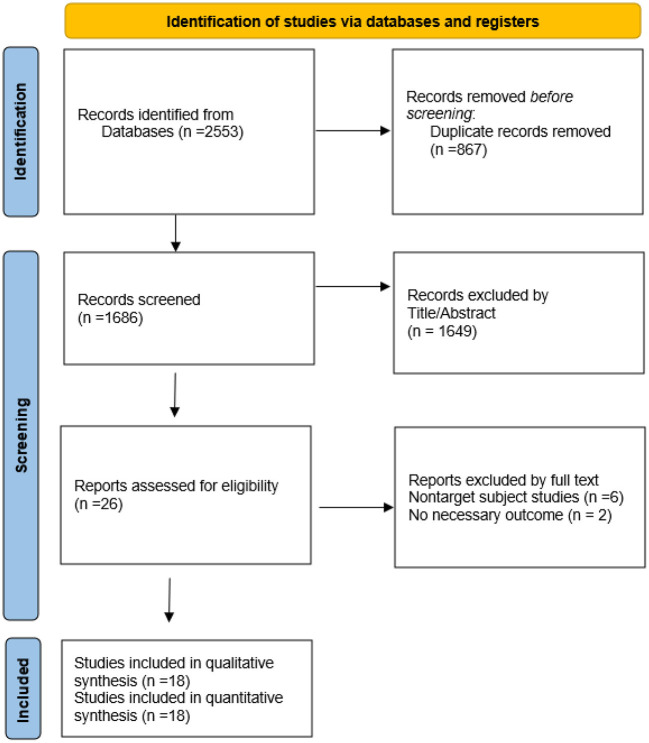
PRISMA flow diagram.

**Figure 2:**
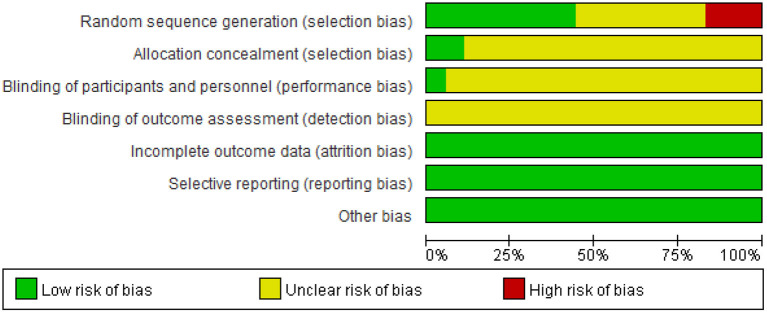
Risk-of-bias summary.

**Figure 3:**
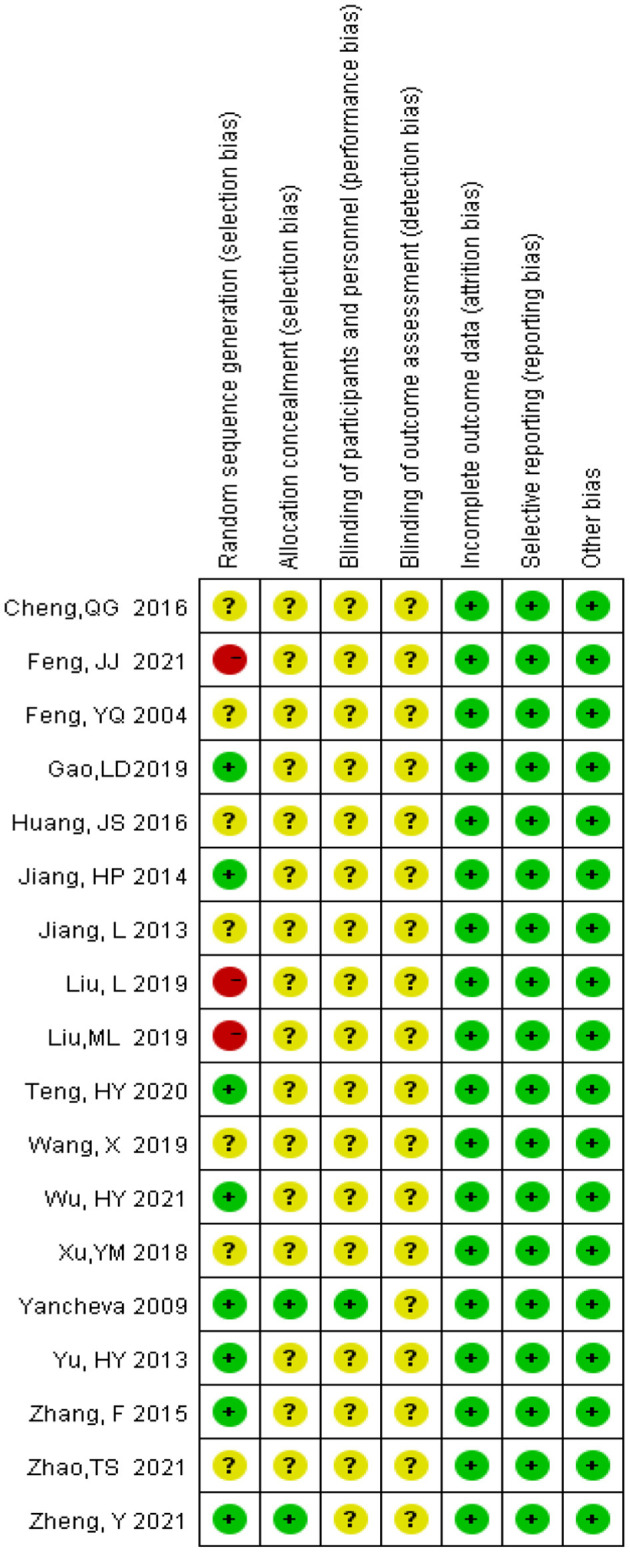
Risk-of-bias graph.

As shown in Figure 1, the electronic databases were searched for studies on ginkgo combined with donepezil hydrochloride vs. donepezil hydrochloride for the treatment of dementia. A total of 2,553 studies were initially retrieved. Of these, 867 duplicate papers and 1,649 irrelevant studies that did not meet the inclusion criteria were excluded after their titles and abstracts were read, and 8 studies were excluded after reading the full text. Thus, a total of 18 studies were included in the analysis (Feng et al., 2004; Yancheva et al., 2009; Jiang et al., 2013, 2014; Yu et al., 2013; Zhang et al., 2015; Cheng et al., 2016; Huang et al., 2016; Xu et al., 2018; Gao et al., 2019; Liu L. et al., 2019; Liu M. L. et al., 2019; Wang, 2019; Teng et al., 2020; Feng, 2021; Wu et al., 2021; Zhao et al., 2021; Zheng et al., 2021). The qualities of the studies reported in these studies were then evaluated. Figures 2, 3 show the graphs of the risk of bias summary and the risk of bias ratio, respectively. The results of the quality assessment showed that the studies reported in the studies are of relatively high quality.

### 3.2. Studies' characteristics

The basic characteristics of the included studies are in Table 1. Eighteen involved trials were included, which include 1,642 participants. Of these, 842 participants were in the experimental group (ginkgo biloba preparations combined with donepezil hydrochloride) and 800 participants were in the control group (donepezil), published from 2004 to 2022. The size of samples included in the study varies from 140 cases to 40 cases. There were seventeen trials, six trials, nine trials, three trials, two trials, and five trials, respectively, which reported the curative effect, MMSE score, ADL score, HDS score, MoCA score, and adverse events. In addition, the treatment course ranges from 12 weeks to 3 months.

**Table 1:** Basic characteristics of the included studies.

**References**	**Sample size**		**Age**		**Outcome**	**Treatment course**
	**Experimental group**	**Control group**	**Experimental group**	**Control group**		
Cheng et al. (2016)	30	30	74.13 ± 4.93	72.87 ± 4.83		3 months
Feng (2021)	64	64	65.87 ± 5.64	65.63 ± 5. 61		6 months
Feng et al. (2004)	22	20	79 ± 6	70 ± 7		12 weeks
Gao et al. (2019)	58	58	71.0 ± 8.3	70.6 ± 8.8		9 months
Huang et al. (2016)	40	40	66.9 ± 4.1	66.1 ± 3.9		3 months
Jiang et al. (2014)	40	40	66.9 ± 4.1	66.1 ± 3.9		3 months
Jiang et al. (2013)	31	29	65.90 ± 5.29	66.38 ± 5.16.		3 months
Liu L. et al. (2019)	50	50	73.32 ± 10.15	73.40 ± 11.19		4 months
Liu M. L. et al. (2019)	50	50	70.02 ± 2.55	70.10 ± 2.63		6 months
Teng et al. (2020)	59	56	68.59 ± 7.03	67.84 ± 7.41		3 months
Wang (2019)	40	40	64.05 ± 10.12	63.38 ± 10.62		6 months
Wu et al. (2021)	49	49	67.4 ± 0.9			6 months
Xu et al. (2018)	38	38	74.15 ± 4.93	72.85 ± 4.83		6 months
Yancheva et al. (2009)	32	33	68 ± 9	66 ± 8		12 weeks
Yu et al. (2013)	52	50	69.94 ± 10.48	70.15 ± 9.27.		24 weeks
Zhang et al. (2015)	30	30	68.5 ± 5.3	68.2 ± 5.6		4 months
Zhao et al. (2021)	70	70	73.32 ± 10.15	73.40 ± 11.19		3 months
Zheng et al. (2021)	50	50	71.3 ± 8.2	71.1 ± 7.6		6 months

Efficacy, MMSE, Mini-mental State Examination; ADL, Activity of Daily Living Scale; HDS, Hasegawa dementia scale; MoCA, Montreal Cognitive Assessment; adverse events.

### 3.3. Methodology quality

The methodological quality of these studies was evaluated using Cochrane Collaboration's tool for assessing the risk of bias. For the random sequence generation, eight trials were judged to be low risk of bias while two trials were judged to be high risk. For allocation concealment only two trials were judged to be low risk of bias, other trials were judged to be at an unclear risk of bias. For blinding of participants and personnel, only one trial explains the specific double-blind method and double-blind simulation technique, and other trials were judged to be an unclear risk due to lacking specific intervention details. Three trials have given the reasons for the incomplete outcome data.

### 3.4. Meta-analysis results

#### 3.4.1. Clinical effectiveness rate

**Figure 4:**
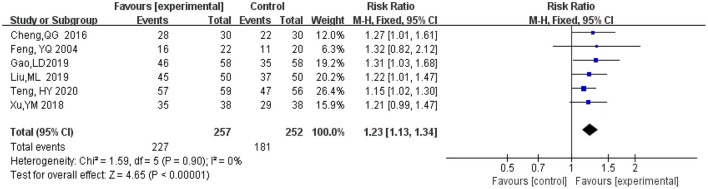
Meta-analysis of clinical effectiveness rate.

A total of six studies (Feng et al., 2004; Cheng et al., 2016; Xu et al., 2018; Gao et al., 2019; Liu M. L. et al., 2019; Teng et al., 2020) included statistical analysis of clinical effectiveness rate after treatment with ginkgo combined with donepezil hydrochloride vs. donepezil hydrochloride in patients with dementia. The judgment of clinical effectiveness is mainly based on the improvement of the MMSE score (when the MMSE score improves from 4 or 1 to 3, the clinical efficacy is significant or improved, and the total clinical effectiveness is equal to (significant effect + improvement) (An, 2016). There was a total of 509 participants in these studies, 257 in the experimental group (ginkgo combined with donepezil hydrochloride), of which 227 participants' clinical effectiveness was valid, and 252 in the control group (donepezil hydrochloride), of which 181 participants' clinical effectiveness was valid. Meta-analysis was carried out using RR as the effect size. The Q-test for heterogeneity revealed heterogeneity in the effect sizes (*I*^2^ = 0%, *P* = 0.90), indicating that there was no heterogeneity between studies. Therefore, the fixed effects model and M-H method were used for the analysis. The results of the meta-analysis indicated that the effectiveness recorded in the experimental group was significantly better than that in the control group after months 3 to 9 months of intervention (RR = 1.23, 95% CI 1.13, 1.34, *P* < 0.00001) (Figure 4).

#### 3.4.2. Mini-Mental State Examination scores

**Figure 5:**
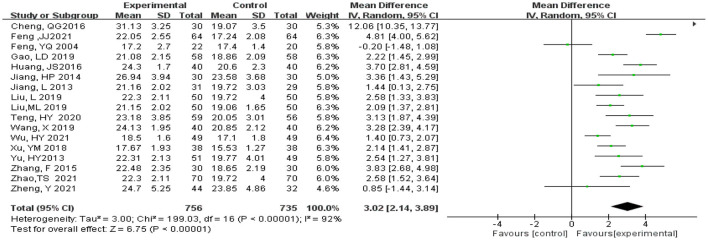
Meta-analysis of Mini-Mental State Examination scores.

Statistical analysis of MMSE scores was conducted in 17 studies (Feng et al., 2004; Jiang et al., 2013, 2014; Yu et al., 2013; Zhang et al., 2015; Cheng et al., 2016; Huang et al., 2016; Xu et al., 2018; Gao et al., 2019; Liu L. et al., 2019; Liu M. L. et al., 2019; Wang, 2019; Teng et al., 2020; Feng, 2021; Wu et al., 2021; Zhao et al., 2021; Zheng et al., 2021). Total of 1,493 subjects, 758 subjects in the experimental and 735 subjects in the control groups, were included in these studies. Meta-analysis was carried out using MD as the effect size. The *Q*-test for heterogeneity revealed heterogeneity in the effect sizes (*I*^2^ = 92% > 50%, *P* < 0.00001), indicating that high heterogeneity existed between studies; that is, homogeneity was relatively poor. Therefore, the random effects model and M-H method were used for analysis. The meta-analysis results indicated that the experimental group had significantly higher MMSE scores than the control group (MD = 3.02, 95% CI 2.14, 3.89, *P* < 0.00001) (Figure 5).

#### 3.4.3. Sensitivity analysis

**Figure 6:**
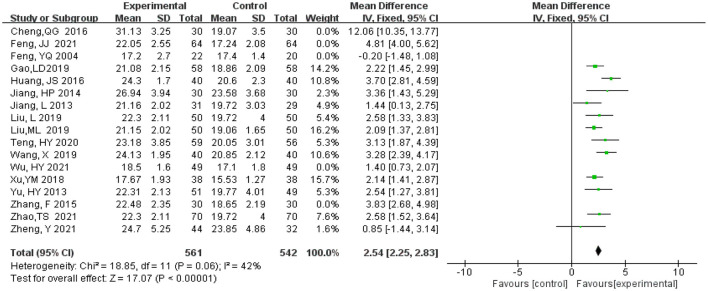
Meta-analysis of sensitivity analysis of MMSE scores.

MMSE scores are highly heterogeneous, therefore, literature elimination and subgroup analysis are adopted to find the source of heterogeneity. After five studies were gradually eliminated (Feng et al., 2004; Zhang et al., 2015; Cheng et al., 2016; Feng, 2021; Wu et al., 2021), the heterogeneity decreased from 92 to 42%. There is little change in the combined effect size ranges from (MD = 3.02, 95% CI 2.14, 3.89, *P* < 0.00001) to (MD = 2.54, 95% CI 2.25, 2.83, *P* < 0.00001) (Figure 6), which indicates that heterogeneity does not have much influence on the research results and our result that experimental group had significantly higher MMSE scores than the control group.

#### 3.4.4. Subgroup analysis

**Figure 7:**
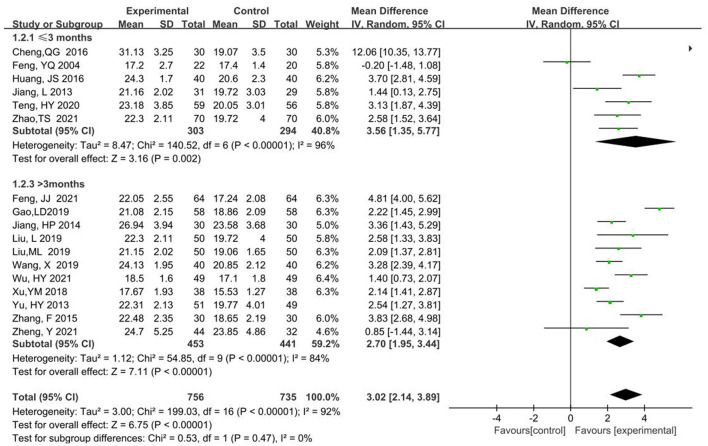
Meta-analysis of subgroup analysis of MMSE scores.

MMSE scores were analyzed in subgroups according to two kinds of treatment courses < 3 months or equal to 3 months, and longer than 3 months. Subgroup 1 ( ≤ 3 month) had a total of 597 participants, 303 in the experimental and 294 in the control groups, respectively (MD = 3.56, 95% CI 1.35, 5.77, *P* < 0.00001). Subgroup 2(>3 month) had a total of 894 participants, 453 in the experimental and 441 in the control groups (MD = 2.70, 95% CI 1.95, 3.44, *P* < 0.00001). For the two subgroups, the combined effects model was chosen as the random effects model. The *P*-value was >0.05 and heterogeneity decreased from 92 to 0% in the combined analysis. Thus, the differences between the two subgroups were not statistically significant and the difference in treatment course was the major source of heterogeneity (Figure 7).

#### 3.4.5. Activity of Daily Living Scale score

**Figure 8:**
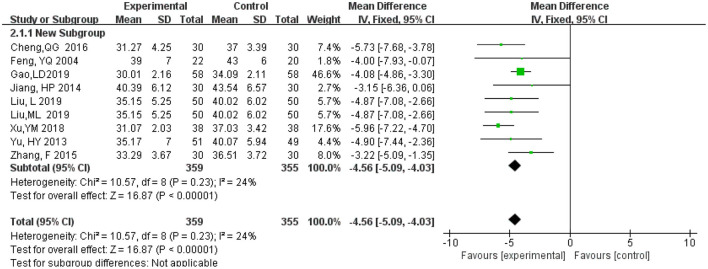
Meta-analysis of ADL.

A total of 715 participants, of these 359 in the experimental group and 356 in the control group in nine studies (Feng et al., 2004; Yu et al., 2013; Jiang et al., 2014; Zhang et al., 2015; Cheng et al., 2016; Xu et al., 2018; Gao et al., 2019; Liu L. et al., 2019; Liu M. L. et al., 2019). Meta-analysis was carried out using MD as the effect size. The *Q*-test for heterogeneity showed heterogeneity in the effect sizes (*I*^2^ = 24%, *P* = 0.23), indicating that homogeneity was relatively good between studies. Thus, the random effects model and M-H method were used for analysis The results showed that the experimental group had a significantly lower ADL score than the control group (MD = −4.56, 95% CI −5.09, −4.03, *P* < 0.00001) (Figure 8).

#### 3.4.6. Hasegawa Dementia Scale

**Figure 9:**
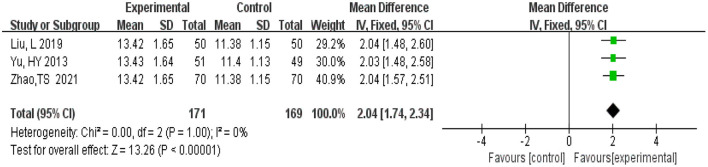
Meta-analysis of Hasegawa Dementia Scale.

A total of 340 participants were from three studies (Yu et al., 2013; Liu L. et al., 2019; Zhao et al., 2021). There were 171 in the experimental group and 169 patients in the control group. Meta-analysis was conducted using MD as the effect size. The *Q*-test for heterogeneity revealed heterogeneity in the effect sizes (*I*^2^ = 0%, *P* = 1.00), indicating that no heterogeneity existed between studies, which means homogeneity was relatively good. Therefore, the fixed effects model and M-H method were used for analysis. The results of the meta-analysis indicated that the experimental group had lower Hasegawa Dementia Scale scores than the control group (MD = 2.04, 95% CI 1.74, 2.34, *P* < 0.00001) (Figure 9).

#### 3.4.7. Montreal Cognitive Assessment

**Figure 10:**
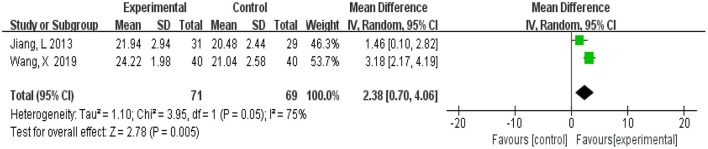
Meta-analysis of Montreal Cognitive Assessment.

A total of 140 participants were from two studies (Jiang et al., 2013; Wang, 2019), 71 in the experimental group and 69 in the control group. Meta-analysis was carried out using MD as the effect size. The *Q*-test for heterogeneity showed heterogeneity in the effect sizes (*I*^2^ = 75%, *P* = 0.05), indicating that there was high heterogeneity between studies. Thus, the random effects model and M-H method were used for analysis. The meta-analysis results showed that patients in the experimental group had significantly better vocabulary memory than the control group (MD = 2.38, 95% CI 0.72, 4.06, *P* = 0.005) (Figure 10).

#### 3.4.8. Adverse events

**Figure 11:**
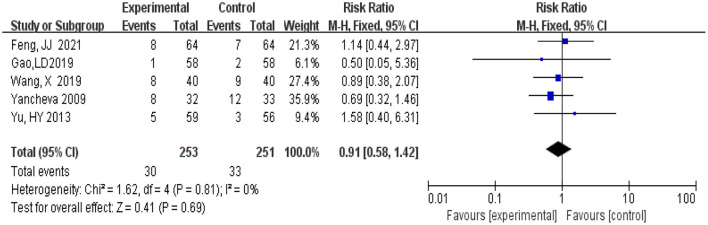
Meta-analysis of adverse events.

A total of 504 participants, 253 in the experimental group and 251 patients in the control group, were included in five studies (Yancheva et al., 2009; Yu et al., 2013; Gao et al., 2019; Wang, 2019; Feng, 2021). Meta-analysis was conducted using RR as the effect size. The *Q*-test for heterogeneity showed heterogeneity in the effect sizes (*I*^2^ = 0, *P* = 0.81), the 95 %CI horizontal line of RR in included studies intersects with the invalid vertical line (the abscissa scale is 1), which means that there is no significant difference between the incidence of the experimental group and that of the control group, and it cannot be determined that the incidence of the experimental group is not equal to that of the control group (RR = 0.91, 95% CI 0.58, 1.42, *P* = 0.69) (Figure 11).

### 3.5. Analysis of publication bias

**Figure 12:**
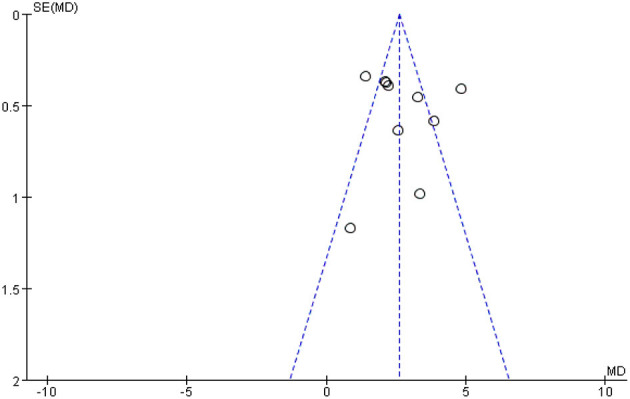
Funnel plot for MMSE scores.

Figure 12 shows a funnel plot of the MMSE scores outcome. The funnel plot indicates that the participants of the studies reported in the included studies are largely within the triangular area and largely symmetrically distributed, while three studies fall outside the confidence interval of the funnel graph, indicating that heterogeneity may be the main reason for the asymmetry of funnel graph, that is, heterogeneity may be an important factor for publication bias, suggesting that there may a certain publication bias in the studies.

### 3.6. Quality of evidence

The GRADE evidence profiles for primary and secondary outcomes are shown in Table 2.

**Table 2:** GRADE evidence profile.

**Certainty assessment**							**No. of patients**		**Effect**		**Certainty**	**Importance**
**No. of studies**	**Study design**	**Risk of bias**	**Inconsistency**	**Indirectness**	**Imprecision**	**Other considerations**	**ginkgo biloba preparations combined with donepezil hydrochloride**	**donepezil**	**Relative (95% CI)**	**Absolute (95% CI)**		
**Clinical effectiveness rate**												
6	Randomized trials	Serious^a^	Not serious	Not serious	Not serious	None	227/257 (88.3%)	181/252 (71.8%)	RR 1.23 (1.13 to 1.34)	165 more per 1,000 (from 93 more to 244 more)	⊕⊕⊕○ Moderate	Critical
**Mini-Mental State Examination scores**												
17	Randomized trials	Serious^a^	Serious^b^	Not serious	Not serious	None	756	735	–	MD 3.02 higher (2.14 higher to 3.89 higher)	⊕⊕○○ Low	Critical
**Barthel index**												
9	Randomized trials	Serious^a^	Not serious	Not serious	Not serious	None	359	355	–	MD 4.56 lower (5.09 lower to 4.03 lower)	⊕⊕⊕○ Moderate	Important
**Hasegawa Dementia Scale**												
3	Randomized trials	Serious^a^	Not serious	Not serious	Serious^c^	None	171	169	–	MD 2.04 higher (1.74 higher to 2.34 higher)	⊕⊕○○ Low	Important
**Montreal Cognitive Assessment**												
2	Randomized trials	Serious^a^	Not serious	Not serious	Serious^c^	None	71	69	–	MD 2.38 higher (0.7 higher to 4.06 higher)	⊕⊕○○ Low	Important

^a^High risk of bias in assigning concealment and blindness.

^b^High heterogeneity.

^c^Small sample size.

## 4. Discussion

### 4.1. Main findings

In this study, we conducted a meta-analysis of 18 studies on the treatment of Alzheimer's disease using ginkgo biloba preparations combined with donepezil hydrochloride vs. donepezil to investigate the effectiveness of ginkgo biloba preparations combined with donepezil hydrochloride. We found that compared to donepezil alone, ginkgo biloba preparations combined with donepezil are more effective in improving MMSE score, ADL score, HDS score, and MoCA score of patients with Alzheimer's disease within 12 to 9 weeks of intervention. However, we haven't found the adverse events difference between ginkgo biloba preparations combined with donepezil and donepezil alone which indicates the safety of ginkgo biloba preparations combined with donepezil hydrochloride is unsure due to the low quality.

### 4.2. Interpretation of findings

Previous meta-analysis shows that Ginkgo biloba leaves have potential benefits in treating mild cognitive function and AD patients' cognitive function, daily living ability, and overall evaluation, but it has not explained the efficacy and safety of Ginkgo biloba leaves in treating mild cognitive impairment and AD (Yang et al., 2016). Recently, another network meta-analysis further confirmed that EGb with a dose of 240 mg may be the best intervention measure for acceptability and safety while a high risk of other bias was noted in six studies in their network meta-analysis (Zhang et al., 2020). Our meta-analysis included the recent high-quality randomized controlled studies, and the outcomes selected the classic dementia screening tools such as MMSE, HDS, MoCA, and ADL, which can better reflect the effect of ginkgo biloba preparations combined with donepezil on improving cognitive function and ADL. At the same time, our meta-analysis reviewed the effectiveness and safety of ginkgo biloba preparations and found a statistically significant conclusion that was not found in previous studies: ginkgo biloba preparations combined with donepezil can better improve patients' cognitive function and ADL, but the safety needs further verification.

Ginkgo biloba as a traditional Chinese medicine has a long history of medication, which contains many active ingredients, such as ginkgolides, bilobalide, and flavonoids (Singh et al., 2019). These ingredients make ginkgo have strong antioxidant and free radical scavenging. Beta-amyloid hypothesis, as the main pathogenesis of AD, holds that Aβ deposition and its neurotoxicity are the main causes of cognitive dysfunction in patients. Several studies showed that EGb protects against Aβ-induced neurotoxicity by obstruction of Aβ-induced events, such as glucose uptake, and reactive oxygen species (ROS) accumulation, it is reported that EGb inhibits the production of Aβ in the brain by lowering the levels of circulating free cholesterol, as AβPP processing and amyloidogenic are supposed to be affected by free circulating and intracellular cholesterol levels (Shi et al., 2010; Singh et al., 2019; Nowak et al., 2021). Colciaghi et al. (2004) investigated the effect of EGb761 on the amyloid precursor protein (APP) metabolism by both *in vitro* and *in vivo* model animal trials and demonstrated that EGb761 increases alphaAPPs while not accompanied by a modification of either APP forms or alpha-secretase expression, which means EGb has an effect on decreasing the levels of Aβ and its neurotoxicity. Additionally, mitochondria function disorder and Apoptosis were considered as the pathological changes seen in AD (Oliver and Reddy, 2019). EGb761 has been suggested to have protective effects on mitochondria, due to its antioxidant effects, as the mitochondrial respiratory chain is both the major target and the major source of ROS (Chen et al., 2019). The flavonoid fraction of EGb761 may be partly responsible for its anti-apoptotic properties. As for possible mechanisms underlying its anti-apoptotic action. EGb761 are multifactorial and may act synergistically upon multiple intracellular signaling pathways involved in apoptosis. The antioxidant effect of flavonoids may reduce the formation of ROS by increasing the activity of the cytochrome P-450 enzyme system and inhibiting the release of peroxide anions. Other components Ginkgolide B It plays an important role in scavenging free radicals and antioxidation (Nowak et al., 2021). The above pharmacological mechanism of ginkgo biloba extract well-supports our research findings that ginkgo biloba preparations combined with donepezil can better improve patients' cognitive function.

### 4.3. Advantages and limitations

This meta-analysis conducts a comprehensive evaluation of the effectiveness of ginkgo biloba preparations combined with donepezil hydrochloride and indicates that ginkgo biloba preparations combined with donepezil can better improve patients' cognitive function and ADL, which is helpful for the application and popularization of ginkgo biloba preparations in AD and provides more ideas for drug research of AD. This study has some predominancies. We register on PROSPRO platform in advance to ensure the transparency and scientific of meta-analysis. Secondly, in order to make the research results more reliable, we have included as many RCTs on the combination of ginkgo biloba preparations and donepezil as possible, and comprehensively analyzed the sources of heterogeneity and bias. This study also has some limitations. Seventeen included studies have high heterogeneity in MMSE scores, which may arise from differences between follow-up RCTs, differences in doses administered, and differences in patient characteristics. Finally, differences in the experience of the physicians in each study may also result in some clinical bias.

## 5. Conclusion

There was a significant difference between ginkgo biloba preparations combined with donepezil and donepezil alone, ginkgo biloba preparations combined with donepezil has a better effect on improving the activities of daily living, a cognitive function such as MMSE and MoCA, However, we have not found the difference in adverse events. High-quality and large-sample multicenter randomized controlled studies are needed to verify it.

## Data availability statement

The original contributions presented in the study are included in the article/Supplementary material, further inquiries can be directed to the corresponding author.

## Author contributions

BW and DL conceived the study. JM and SG collected the data and drafted the manuscript. YL revised the manuscript and language. XW conducted the subgroup analysis. All authors have read and approved the manuscript.
