# Supplementary material for: Effectiveness and safety of ginkgo biloba preparations in the treatment of Alzheimer's disease: A systematic review and meta-analysis
Source: Front Aging Neurosci. 2023 Mar 7;15:1124710. doi: 10.3389/fnagi.2023.1124710 (PMC10028084; doi:10.3389/fnagi.2023.1124710)
Supplement: Supplementary file 1 [file Table_1.docx]

**The retrieval strategies and retrieval results of each database are shown in Tables 1-3.**

Table 1: PubMed

| No. | Content | Result |
| --- | --- | --- |
| #1 | "Alzheimer Disease"[MeSH Terms] | 113,470 |
| #2 | "alzheimer disease"[Title/Abstract] OR "alzheimer*"[Title/Abstract] OR "alzheimer dementia"[Title/Abstract] OR ("alzheimer*"[All Fields] AND "disease"[Title/Abstract]) OR "alzheimer type dementia"[Title/Abstract] | 184,538 |
| #3 | #1 OR #2 | 195,669 |
| #4 | "extract of ginkgo biloba"[Title/Abstract] OR "ginkgo leaf"[Title/Abstract] OR "ginkgo biloba extract"[Title/Abstract] OR "extract of ginkgo"[Title/Abstract] OR "biloba leaves injection"[Title/Abstract] OR "Egb"[Title/Abstract] OR "Ginaton"[Title/Abstract] | 2,276 |
| #5 | #3 AND #4 | 221 |

Table 2: Embase

| No. | Content | Result |
| --- | --- | --- |
| #1 | 'alzheimer disease'/exp | 233,944 |
| #2 | 'alzheimer disease':ab,ti OR alzheimer*:ab,ti OR 'alzheimer dementia':ab,ti OR 'alzheimer* disease':ab,ti OR 'alzheimer type dementia':ab,ti | 234,773 |
| #3 | #1 OR #2 | 280,833 |
| #4 | 'extract of ginkgo biloba':ab,ti OR 'ginkgo leaf':ab,ti OR 'ginkgo biloba extract':ab,ti OR 'extract of ginkgo':ab,ti OR 'biloba leaves injection':ab,ti OR egb:ab,ti OR ginaton:ab,ti | 3,222 |
| #5 | #3 AND #4 | 287 |

Table 3: Cochrane Library

| No. | Content | Result |
| --- | --- | --- |
| #1 | (Alzheimer disease):ti,ab,kw OR (Alzheimer*):ti,ab,kw OR (Alzheimer dementia):ti,ab,kw OR (Alzheimer* disease):ti,ab,kw OR (Alzheimer type dementia):ti,ab,kw | 12,845 |
| #2 | (extract of Ginkgo biloba):ti,ab,kw OR (ginkgo leaf):ti,ab,kw OR (Ginkgo biloba extract):ti,ab,kw OR (extract of Ginkgo):ti,ab,kw OR (biloba leaves injection):ti,ab,kw | 766 |
| #3 | (Egb):ti,ab,kw OR (Ginaton):ti,ab,kw | 304 |
| #4 | #2 OR #3 | 839 |
| #5 | #1 AND #4 | 87 |
